# Supplementary material for: Mechanisms of iron- and O2-sensing by the [4Fe-4S] cluster of the global iron regulator RirA
Source: eLife. 2019 Sep 17;8:e47804. doi: 10.7554/eLife.47804 (PMC6748827; doi:10.7554/eLife.47804)
Supplement: Supplementary file 1. [file elife-47804-supp1.docx]

**Supplementary File 1**

**Predicted and observed masses for apo- and cluster-bound forms of RirA.**

| **RirA species** | **Predicted mass^a^ (Da)** | **Observed mass^b^ (Da)** | **ΔMass^c^**  **(Da)** | **^34^S Isotope**  **shift (Da)** |
| --- | --- | --- | --- | --- |
| **Monomeric** | | | |  |
| Apo | 17442 | 17441 | -1 | 0 |
| [4Fe-4S]^2+^ | 17792 | 17792 | 0 | +8 |
| [4Fe-3S]^4+^ | 17758 | 17758 | 0 | +6 |
| [3Fe-4S]^1+^ | 17737 | 17736 | -1 | +8 |
| [3Fe-3S]^3+^ | 17703 | 17703 | 0 | +6 |
| [3Fe-2S]^5+^ | 17669 | 17668 | -1 | +4 |
| [3Fe-S]^7+^ | 17635 | 17635 | 0 | *+2* |
| [2Fe-2S]^2+^ | 17616 | 17615 | -1 | +4 |
| [2Fe-S]^4+^ | 17582 | 17582 | 0 | +2 |
| 2Fe^2+^ | 17550 | 17550 | 0 | 0 |
| **Dimeric** | | | |  |
| [4Fe-4S]^2+^/[4Fe-4S]^2+^ | 35584 | 35584 | 0 |  |
| [3Fe-4S]^1+^/[4Fe-4S]^2+^ | 35529 | 35529 | 0 |  |
| [3Fe-4S]^1+^/[3Fe-4S]^1+^ | 35474 | 35475 | 0 |  |
| [2Fe-2S]^2+^/[3Fe-4S]^1+^ | 35353 | 35353 | 0 |  |
| [2Fe-2S]^2+^/[2Fe-2S]^2+^ | 35232 | 35234 | +2 |  |
| Apo/[2Fe-2S]^2+^ | 35058 | 35058 | 0 |  |
| Apo/Apo | 34884 | 34882 | -2 |  |

^a^The predicted mass depends on the cluster/cluster fragment charge because binding is assumed to be charge compensated (43,44). Cluster charge states are as observed previously (43).

^b^The average observed mass is derived from at least four independent experiments, with standard deviation of ± 1 Da.

^c^The difference between the average observed and predicted masses.
