# Supplementary material for: Mechanisms of iron- and O2-sensing by the [4Fe-4S] cluster of the global iron regulator RirA
Source: eLife. 2019 Sep 17;8:e47804. doi: 10.7554/eLife.47804 (PMC6748827; doi:10.7554/eLife.47804)
Supplement: Supplementary file 2. [file elife-47804-supp2.docx]

**Supplementary File 2**

**Rate constants resulting from global fit of experimental ESI-MS data for RirA dimer using the model shown in Figure 10 – figure supplement 1.**

| Reaction step | Rate constant (min^-1^)^a,b^ | Reaction step |
| --- | --- | --- |
|  | Anaerobic |  |
| *k_1_* | 0.31 ± 0.01 | [4Fe-4S]/[4Fe-4S] → [3Fe-4S]/[4Fe-4S] + Fe |
| *k_-1_* | 4.67 ± 0.43× 10^3^ | [3Fe-4S]/[4Fe-4S] + Fe → [4Fe-4S]/[4Fe-4S] |
| *k_2_* | 0.31 ± 0.01 | [3Fe-4S]/[4Fe-4S] → [3Fe-4S]/[3Fe-4S] + Fe |
| *k_-2_* | 4.67 ± 0.43× 10^3^ | [3Fe-4S]/[3Fe-4S] + Fe → [3Fe-4S]/[4Fe-4S] |
| *k_3_* | 0.09 ± 0.01 | [3Fe-4S]/[3Fe-4S] → [2Fe-2S]/[3Fe-4S] |
| *k_4_* | 0.09 ± 0.01 | [2Fe-2S] [3Fe-4S] → [2Fe-2S]/[2Fe-2S] |
| *k_5_* | 0.10 ± 0.03 | [2Fe-2S]/[2Fe-2S] → apo/[2Fe-2S] |
| *k_6_* | 0.10 ± 0.03 | apo/[2Fe-2S] → apo/apo |

^a^With the exception of *k*_-1_, which is a second order rate constant with units of M^-1^ min^-1^.

^b^Standard errors are indicated.
